# PEGylation renders carnosine resistant to hydrolysis by serum carnosinase and increases renal carnosine levels

**DOI:** 10.1007/s00726-024-03405-6

**Published:** 2024-07-04

**Authors:** Shiqi Zhang, Guang Yang, Qinqin Zhang, Yuying Fan, Mingna Tang, Liuhai Shen, Dongchun Zhu, Guiyang Zhang, Benito Yard

**Affiliations:** 1 Department of Endocrinology, The first affiliated hospital of Anhui Medical University, Hefei, 230022 China; 2 Department of Pharmacy, The first affiliated hospital of Anhui Medical University, Hefei, 230022 China; 3 Department of Nuclear Medicine, Provincial Peoplès Hospital, Anhui No. 2 Hefei, 230041 China; 4 Department of Pharmacology, School of Basic Medical Sciences, Anhui Medical University, Hefei, 230032 China; 5 Vth Department of Medicine (Nephrology/Endocrinology/Rheumatology), University Medical Center Mannheim, University of Heidelberg, 68167 Mannheim, Germany

**Keywords:** L-carnosine, Diabetic nephropathy, PEGylated carnosine, Pharmacokinetics

## Abstract

Carnosine’s protective effect in rodent models of glycoxidative stress have provided a rational for translation of these findings in therapeutic concepts in patient with diabetic kidney disease. In contrast to rodents however, carnosine is rapidly degraded by the carnosinase-1 enzyme. To overcome this hurdle, we sought to protect hydrolysis of carnosine by conjugation to Methoxypolyethylene glycol amine (mPEG-NH_2_). PEGylated carnosine (PEG-car) was used to study the hydrolysis of carnosine by human serum as well as to compare the pharmacokinetics of PEG-car and L-carnosine in mice after intravenous (IV) injection. While L-carnosine was rapidly hydrolyzed in human serum, PEG-car was highly resistant to hydrolysis. Addition of unconjugated PEG to carnosine or PEG-car did not influence hydrolysis of carnosine in serum. In mice PEG-car and L-carnosine exhibited similar pharmacokinetics in serum but differed in half-life time (t_1/2_) in kidney, with PEG-car showing a significantly higher t_1/2_ compared to L-carnosine. Hence, PEGylation of carnosine is an effective approach to prevent carnosine degradations and to achieve higher renal carnosine levels. However, further studies are warranted to test if the protective properties of carnosine are preserved after PEGylation.

## Introduction

L-carnosine, a natural histidine-containing dipeptide (HCD), is abundantly present in excitable tissues such as skeletal muscle and the central nervous system (CNS), while in non-excitable tissues carnosine levels are much lower(Stede et al. 2023). Although the physiological role of carnosine and other HCDs in kidney and other non-excitable tissues is subject of ongoing discussion, over the past decade carnosine has been reported to exert protective properties in an array of different disease models (Zhao et al. 2017; Busa et al. 2022; Yang et al. 2023; Alsheblak et al. 2016; Caruso et al. 2020; Sun et al. 2017; Miceli et al. 2018; Peters et al. 2020; Liu et al. 2020). Prompted by the finding that the carnosinase 1 gene (*CNDP1*) is a susceptibility locus for developing diabetic kidney disease (DKD) in patients with type 2 diabetes, various studies have reported possible mechanisms by which carnosine may convey protection against DKD (Liu et al. 2020; Zhu et al. 2021; Boldyrev et al. 2013). Also, small clinical trials using carnosine supplementation in obese patients have reported promising results (Courten et al. 2016; Houjeghani et al. 2018; Menon et al. 2018). However, detection of serum carnosine after oral supplementation is only found in individuals with low carnosinase activity (Everaert et al. 2012), suggesting that the efficacy of carnosine supplementation might be hampered by the action of the carnosine hydrolyzing enzyme serum carnosinase 1. Hence, carnosinase-resistant carnosine derivatives, e.g. D-carnosine-octylester (Vistoli et al. 2009) and, FL-926-16 ((2 S)-2-(3-amino propanolamino)-3-(1 H-imidazol-5-yl) propanol) (Iacobini et al. 2018), were developed and showed a good therapeutic efficacy in rodent models.

Polyethylene glycol (PEG) is an important excipient for drug delivery as it has no immunogenicity and has a high chemical stability and tolerability. These properties explain its wide use in different pharmaceutical formulations. Moreover, PEG based pharmaceutical formulations are approved by the US Food and Drug Administration (FDA) as drug delivery excipient in parenteral, topical, ophthalmic, oral and rectal administrations (Gullapalli and Mazzitelli 2015; D’Souza and Shegokar 2016; Veronese and Pasut 2005). PEGylation is mainly designed to improve drug solubility, stability and permeability (Abet et al. 2017) with linear PEG being the most widely used for drug delivery (Kadajji and Betageri 2011). Dependent of its molecular weight (MW), the physical states of PEG vary from clear liquids, soft solids to hard crystalline solids for PEG with low (100–700 kDa) intermediated (1000–2000 kDa) and high MW (> 2000 kDa) (Thomas et al. 2014). Even so, PEG has high solubility in most of the organic as well as inorganic solvents due to its high polarity caused hydrophilicity (Pasut and Veronese 2012). In most cases, PEG 2000 (kDa) has been the standard in the preparation of PEGylated liposomes (Saw et al. 2015; Luk et al. 2012; Nie et al. 2012; Qhattal et al. 2014).

Based on the advantages of PEG we sought to assess if PEGylation of carnosine (PEG-car) using methoxypolyethylene glycol amine (mPEG-NH_2_) would render carnosine resistant to hydrolysis by carnosinase. We also performed a limited pharmacokinetic study to compare serum and renal t_1/2_ for PEG-car and L-carnosine after an intravenous bolus injection in mice.

## Materials and methods

### Synthesis of PEGylated carnosine

**Fig. 1:**
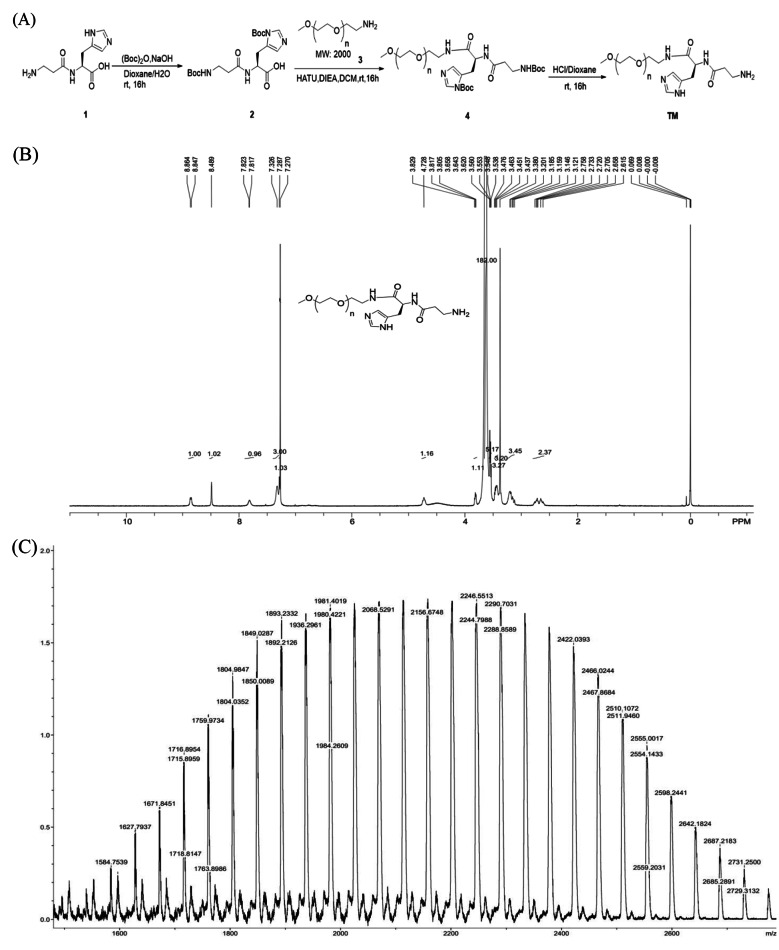
Synthesized process of PEG-car (**A**) and structural characterization of PEG-car by ^1^H-NMR spectrum (**B**) and MALDI-TOF spectrum (**C**)

Synthesis of mPEG-carnosine is shown in Fig. 1A and consisted of a sequential BOC-anhydride and amidation reactions. In brief, for the BOC-carnosine preparation, L-carnosine (10 g, 1 eq) (Sigma-Aldrich, St. Louis, MO, USA), NaOH (1.95 g, 1.1 eq), 100 mL of 1,4 dioxane (Hefei Baierdi Chemical Technology Co. Ltd, Hefei, China) and 100 mL deionized water were mixed in a round-bottom flask. BOC-anhydride (21 g, 2.2 eq) (Hefei Baierdi Chemical Technology Co. Ltd, Hefei, China), was added to the flask and continuously mixed overnight (16 h) at 0℃. Hereafter, the mixture was concentrated up to dryness and the solid residue was extracted with 150 mL ethyl acetate followed by washing with 100 mL deionized water and 50 mL brine. The crude product was loaded on silica gel column (100–200 mesh, petroleum ether: ethyl acetate 5:1 as developing solvent). After elution, 12.5 g Boc-carnosine was yielded (yield, 66.3%).

For the Boc-carnosine-mPEG production, Boc-carnosine (3 g, 1 eq), linear chain methoxypolyethylene glycol amine with a molecular weight of 2000 kDa (mPEG-NH_2,_ 21.1 g, 1.5 eq) (Ponsure Company, Shanghai, China), O-(7-Azabenzotriazol-1-yl)-N, N, N’, N’-tetramethyluronium hexafluorophosphate (HATU, 4 g, 1.5 eq) (Sigma-Aldrich, St. Louis, MO, USA), N, N-Diisopropylethylamine (DIPEA, 1.8 g, 2 eq) (Sigma-Aldrich, St. Louis, MO, USA) were dissolved in 50 mL *N, N*-Dimethylformamide (DMF) (Sigma-Aldrich, St. Louis, MO, USA) and stirred at room temperature for 16 h under nitrogen. The purity (> 95%) of mPEG-NH_2_ was demonstrated by ^1^H-NMR. HATU was used as dehydrating agent to activate carboxyl and promoted the formation of an ester bond. As an alkaline reagent, DIPEA was used as nucleophilic reagent and acid binding agent. On the next day, the mixture was concentrated and extracted by 150 mL ethyl acetate and then washed twice with 100 mL deionized water and twice with 50 mL brine. Afterwards, the crude products were loaded and purified on silica gel column (100–200 mesh, petroleum ether: ethyl acetate 10:1 as developing solvent). After elution, 15 g Boc-carnosine-mPEG was yielded (yield, 88.8%).

The eventual step is the conjugation of PEG and L-carnosine. In this case, Boc-carnosine-mPEG (15 g, 1 eq) was dissolved in 20 mL dioxane and then a mixture of 50 mL dioxane and hydrochloric acid was added. Hereafter, the mixture was concentrated in vacuum and subsequently loaded and purified on silica gel column (100–200 mesh, Dichloromethane: Methanol 10:1 as developing solvent) to harvest the white final compound, 5.9 g mPEG-carnosine (PEG-car) (yield, 42.9%).

### Chemical characterized analysis of synthesized PEG-car

^1^H-NMR (Nuclear Magnetic Resonance Spectroscopy) was carried out to confirm PEGylation of L-carnosine. PEG-car was first dissolved in DMSO-*d*_*6*_ (Sigma-Aldrich, St. Louis, MO, USA) and analyzed by Bruker AVANCE III HD 400 NMR spectrometer (Bruker, Germany) at 400 MHz. The spectral processing with chemical shifts reported in part per million (ppm). The purity of target conjugate was characterized by Ultra-High Performance Liquid Chromatography Mass Spectrometry (UHPLC-MS; Shimadzu LCMS-2020, Japan). The samples were loaded on a chromatographic column (C18, 100 mm×4.6 mm, 5 μm, Waters Sunfire, USA) and eluted in 13 min using a gradient elution containing 0.1% formic acid (J&K Chemical Ltd. Shanghai, China) in ultrapure water as, eluent A and acetonitrile containing 0.1% formic acid as eluent B. 10% eluent B was held for 2 min and the proportion was increased to 95% within 10 min. Hereafter, 95% eluent B was held for f 2 min and the proportion went back to 10% within 0.01 min. with a flowrate of 1 mL/min The injection volume was 10 µL, the column temperature was maintained at 40 °C. Conjugation of L-carnosine to mPEG-NH_2_ was confirmed by matrix assisted laser desorption/ionization time of flight mass spectrometry (MALDI-TOF; Bruker Daltonics, USA). The samples were dissolved in deionized water and 2,5-dihydroxy-benzoic acid (DHB) was used as assisted matrix.

### Enzymatic hydrolysis by carnosinase-1

The resistance of the PEG-car to the enzymatic hydrolysis by carnosinase-1 was assayed by a previously designed enzymatic activity test (Lenney et al. 1982). Briefly, PEG, PEG-car and L-carnosine (1 mM) respectively were incubated in Tris/HCl buffer (50 mM, pH 7.5) at 30 °C with carnosinase-1 (recombinant Human CN-1, Novoprotein Company, Suzhou, China). Aliquots of each sample taken after 5, 10, 20, 30, 40, 60, 80 min, and the enzymatic reaction was stopped by addition of 50 µL of 1% trichloroacetic acid (TCA). After addition of 50 µL of 5 mg/mL *o*-pthaldialdehyde dissolved in 2 M NaOH and incubation for 30 min at 30 °C, the amount of liberated histidine was quantified by fluorometric assay conducted by a multifunctional enzyme marker reader.

### Kinetic studies of PEG-car or L-carnosine in mice

Six- to eight-week-old male C57BL/6J (20–25 g) mice were purchased from Sino-British SIPPR/BK Lab Animal Ltd (Shanghai, China). After ad libitum feeding for one week, the mice were randomly divided into three groups: the control group (*n* = 3, saline treated), the PEG-car treated group (*n* = 3) and the L-carnosine treated group (*n* = 3). Hereof, each mouse was respectively injected intravenously (i.v.) with saline, PEG-car (1000 mg/kg) or L-carnosine (1000 mg/kg) through the tail vein. The mice were anaesthetized by the inhalation of isoflurane during the injection. Blood samples were collected in EDTA tubes by cardiac puncture at 0, 5, 15, 30, 60, 120, 240 min after the administration of PEG-car or L-carnosine. Sera were obtained by centrifugation of the blood sample at 6800 g for 6 min at 4 ℃. Right after sacrifice, the mice were perfused by phosphate-buffered saline to remove the remaining blood. The brains and kidneys were carefully isolated thereafter. The tissues were stored at − 80 ℃and the sera were stored at − 20 ℃ untill use.

This animal study was approved by the Ethics Committee of Animal Research of Anhui Medical University (Hefei, China, No. LLSC20232088).

### Determination of L-carnosine levels by LC-MS/MS

PEG-car or L-carnosine levels in serum and tissues were determined by liquid chromatography tandem-mass spectrometry (LC-MS/MS). Warfarin (Sigma-Aldrich, St. Louis, MO, USA) was used as internal standard (IS). The entire procedure for sample and standard preparation was carried out on ice. An aliquot of 20 µL serum or homogenized 40 mg tissue were pipetted into centrifuge tube and then 400 µL methanol was added for protein precipitation. After spiking the IS (40 ng for serum and 4 ng for kidney), the samples were vortexed for 1 min and then centrifuged at 18,000 g for 7 min at 4 ℃. The supernatant was used for the assay. The calibration curves were prepared by spiking the known concentrations of PEG-car or L-carnosine into serum or tissue homogenates obtained from untreated mice.

The concentrations were measured by a UHPLC system (Shimadzu LC-40D XS, Japan) coupled with electrospray ionization (ESI) and triple quadrupole-ion trap mass spectrometry (AB SCIEX QTRAP^®^ 6500+, Singapore). PEG-car was performed in ACQUITY UPLC BEH C18 column (2.1 mm×50 mm, 1.7 μm, Waters, USA) with a 0.6 mL/min flow rate of mobile phase using gradient elution of ultrapure water (2mM Ammonium acetate (Sinopharm Chemical Reagent Co., Ltd, Shanghai, China) containing 0.1% formic acid, eluent A) and acetonitrile (2mM Ammonium formate (Sinopharm Chemical Reagent Co., Ltd, Shanghai, China) containing 0.1% formic acid, eluent B). The solvent gradient was changed according to the following program: 0–0.01 min 10%B, 0.01–0.6 min 10-90%B, 0.6–1.2 min 90%B, 1.2–1.21 min 90%-10%B, 1.21–1.5 min 10%B. L-carnosine was performed in ACQUITY UPLC BEH Amide column (2.1 mm×50 mm, 1.7 μm, Waters, USA) with a 0.6 mL/min flow rate of mobile phase using gradient elution of ultrapure water (2mM Ammonium acetate containing 0.1% formic acid, eluent A) and acetonitrile (2mM Ammonium formate containing 0.1% formic acid, eluent B). The solvent gradient was as follows: 0–0.01 min 90%B, 0.01–0.6 min 90%-40%B, 0.6–1.2 min 40%B, 1.2–1.21 min 40-90%B, 1.21–1.5 min 90%B. The injection volume was 5 µL. The QTRAP^®^ 6500 + was operated in positive mode, multiple reaction monitoring (MRM) in unit resolution for Q1 and Q3. For detection of PEG-car, three transitions were set with a Q1 mass of 776.4, and Q3 masses of 109.9 (Collision Energy (CE) 119), 126.8 (CE 51), 707.2 (CE 38). For L-carnosine, four transitions were set with a Q1 mass of 227.3, and Q3 masses of 83.1 (CE 53), 110.0(CE 31), 156.2(CE 21), 210.2(CE 16). Pharmacokinetic parameters were calculated by noncompartmental analysis using a sparse sampling and naïve pooled approach (Phoenix WinNonLin 7.0, Certara, Mountain View, CA, USA).

### Statistical analysis

All data are presented as means ± standard error of means (SEM) unless otherwise stated. Subgroup comparisons were analyzed using one-way ANOVA, followed by Tukey’s or Dunnett’s multiple-comparison test. The analysis was evaluated using GraphPad Prism 9.0 (GraphPad Software, Inc., La Jolla, California); *p* < 0.05 was considered statistically significant in all analyses.

## Results

### Structural characterization and purity of PEG-car

**Fig. 2:**
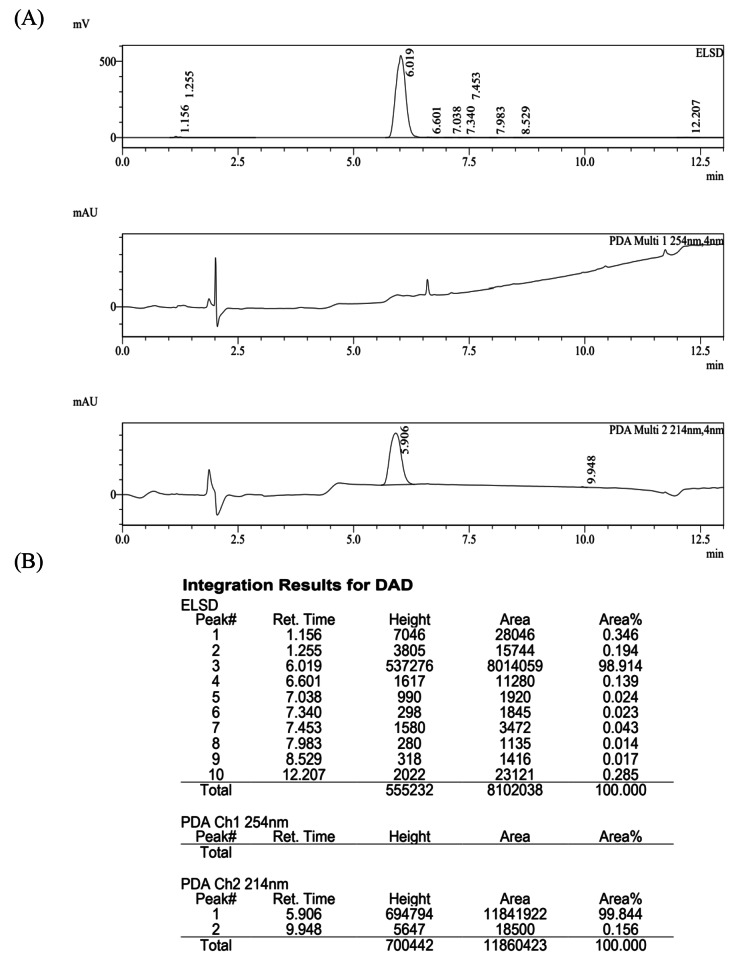
The purity of PEG-car was characterized by HPLC-MS. (**A**) characteristic peaks in HPLC. (**B**) The purity was analyzed by ELSD and PDA.

As depicted in Fig. 1B, ^1^H-NMR (DMSO-*d*_6_, 400 MHz) δ (ppm): 8.85 (d, *J* = 6.8 Hz, 1H), 8.49 (s, 1H), 7.82 (d, *J* = 2.4 Hz, 1H), 7.33 (s, 3 H), 7.29 (s, 1H) were attributed to the protons of imidazole and reactive hydrogen; 4.73 (s, 1H) and 3.82(s, 1H) were attributed to the methylene protons closed to imidazole; 3.66–3.62 (m, 182 H), 3.56–3.54 (m, 5 H), 3.48–3.44 (m, 3 H) and 3.38 (s, 3 H) were attributed to the methylene and terminal methoxy protons of mPEG-NH_2_; 3.20–3.12 (m, 3 H) and 2.76–2.62 (m, 2 H) belonged to the methylene protons of 2-aminoacetamide and the Chiral neutral. As shown in LC-MS (Fig. 2), the purity is greater than 98.9% (ELSD) and 99.8% (PDA, λ = 214 nm). In the MALDI-TOF analysis, the molecular weight (MW) of PEG-car was 2200 kDa, which was close to the theoretical MW as depicted in Fig. 1C. All these characteristic peaks in ^1^H-NMR spectrum as well as MALDI-TOF indicated the successful synthesis of PEG-car.

### PEGylation renders carnosine resistant to carnosinase mediated hydrolysis

**Fig. 3:**
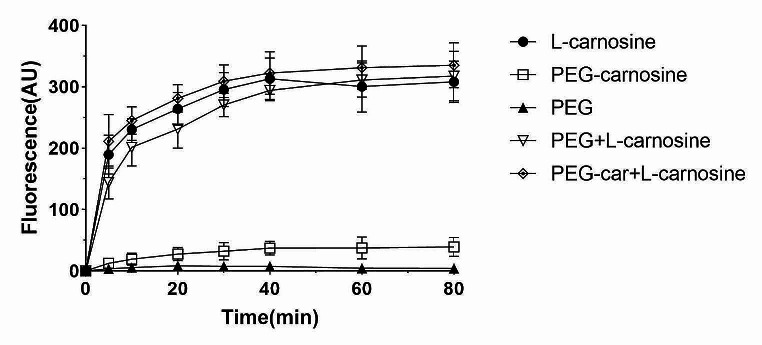
Enzymatic activity towards PEG-car and its parent compounds. The fluorescence intensity (proportional to the histidine content) reported over time due to the action of carnosinase on PEG-car, L-carnosine, PEG, the mixture of the former two and the mixture of the latter two

To assess if PEG-car is resistant to carnosinase mediated hydrolysis, histidine release was assessed. To this end, PEG-car, L-carnosine, mPEG-HN_2_, a mixture of L-carnosine with mPEG-NH_2_ and a mixture of PEG-car with L-carnosine were incubated with recombinant CN-1 for various time intervals and histidine release during each incubation period was determined. As depicted in Fig. 3, no fluorescence was detected in the PEG-NH_2_ sample, while fluorescence increased with time in the carnosine sample. Fluorescence was significantly blunted in the PEG-car sample, approximately six times lower than that of the carnosine sample or the sample containing the mixture of L-carnosine and PEG-NH_2_, in addition, the activity of CN-1 cannot be inhibited by PEG-car.

### Pharmacokinetic of PEG-car or L-carnosine in serum and kidney

**Fig. 4:**
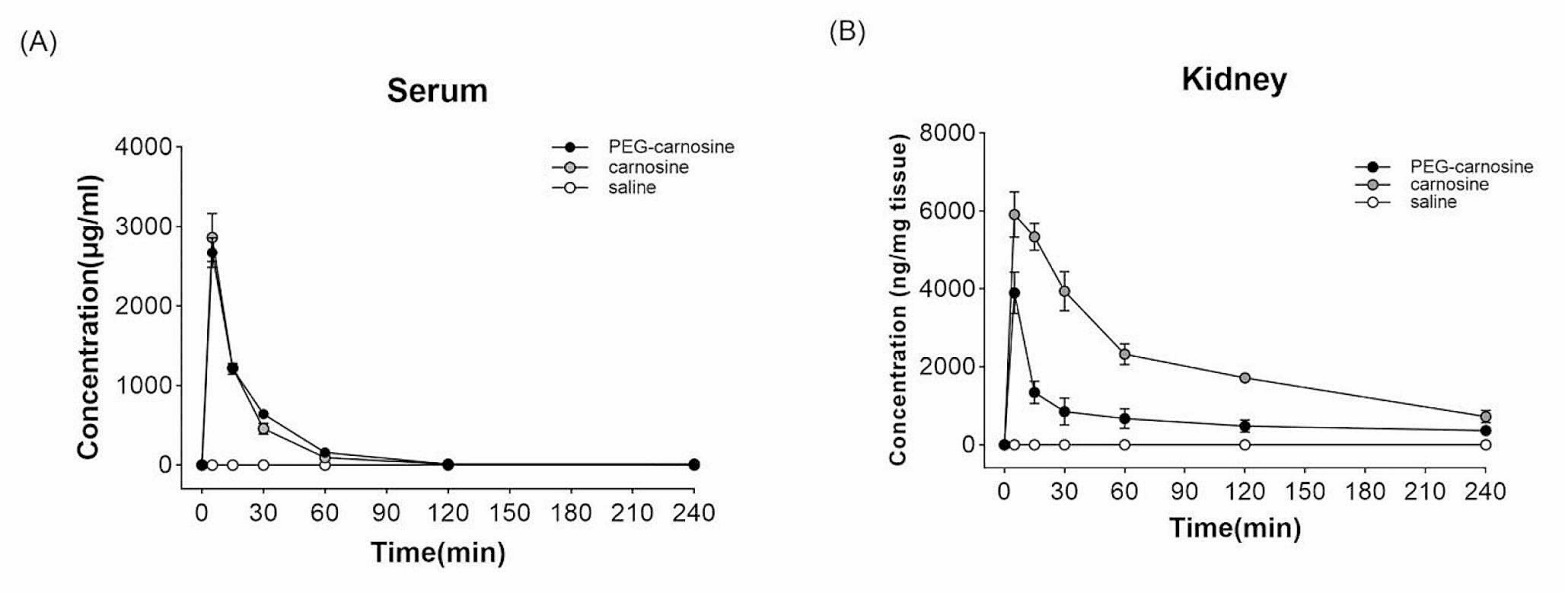
Concentration-time curves of PEG-car and L-carnosine in serum and kidney via intravenous administration in healthy mice; Saline was used as vehicle throughout the study. (**A**) Levels of PEG-car and L-carnosine measured in serum at different time points (0 to 240 min). (**B**) Levels of PEG-car and L-carnosine measured in kidney at different time points (0 to 240 min). Mean ± SEM.

We next assessed t_1/2_ of PEG-car (1000 mg/kg) or L-carnosine (1000 mg/kg) in serum and kidney of mice after a single intravenous injection. Concentrations of PEG-car and L-carnosine were measured by LC-MS/MS and plotted against time (Fig. 4) for calculation of the area under the curve (AUC), peak concentration (Cmax), halve-life (t_1/2_), clearance (CL) and steady state volume of distribution (Vss) for PEG-car and L-carnosine. As shown in Table 1; Fig. 4, with exception of Vss, no differences between the two compounds were observed in serum, while in renal tissue t_1/2_ and Vss of PEG-car were higher compared to L-carnosine.

**Table 1:** Pharmacokinetic analysis of PEG-car and carnosine in serum and kidney

		Half-Life (min)	C_max_ (5 min) (µg/mL)	AUC (µg/mL·min)	CL (mL/min/kg)	Vss (mL/kg)
serum	PEG-car	32	2671	67,730	14.84	294*
	L-carnosine	62	2862	64,871	15.43	606
kidney	PEG-car	168*	3899^b^*	173,340^c^*	3.47^d^*	830^e^*
	L-carnosine	105	5912^b^	518,040^c^	1.59^d^	208^e^

^b^: ng/mg tissue; ^c^: ng/mg tissue·min; ^d^: g/min/kg; ^e^: g/kg

*: *p* < 0.05, compared to L-carnosine group

## Discussion

carnosine is a bioactive peptide, which has the functions of buffering, antioxidation, anti-inflammation, scavenging free radicals, antiaging and preventing metabolic disorders (Kilis-Pstrusinska 2020). However, the potential protective action of this dipeptide in human is hampered by the carnosine hydrolyzing carnosinase enzymes (CN-1 and CN-2). Human *CNDP1* encodes the secreted serum CN-1 with high specificity for L-carnosine, whereas human *CNDP2* encodes tissue and cytosolic CN-2 which is a ubiquitous dipeptidase (Teufel et al. 2003). Both CN-1 and CN-2 can hydrolyze L-carnosine efficiently. Therefore, different derivatives of L-carnosine have been synthesized to retard or prevent carnosine the degradation (Iacobini et al. 2018; Kulikova et al. 2020). This can be accomplished by inhibition of carnosinase activity (Qiu et al. 2019) or by making carnosine derivates that are resistant to carnosinase mediated hydrolysis. PEGylation transforms physicochemical properties of parent molecules like molecular weight, size, hydrophilicity, conformation, steric hindrance, ionic properties and etc. which leads to altered elimination kinetics. This increases in molecular size helps bypass elimination from kidney through glomerular filtration. When the molecular weights are below 20,000 kDa, the clearance by kidney predominates (Yamaoka et al. 1994, 1995).

The spectrum and the final MW of PEG-car as shown in Fig. 1 indicated the successful synthesis, however, with relatively low production ratio (only 42.9%, yield). A more suitable catalyst selection might increase the yield, but this must be proved in the future experiment.

Nevertheless, our study demonstrated that histidine was significantly less released from PEG-car than L-carnosine under the hydrolysis of CN-1 as shown in Fig. 3, which suggested the relatively higher enzymatic stability of PEG-car. In contrast, the simple spike of the PEG solution into L-carnosine did not prevent the breakdown of L-carnosine, which excluded the influence of PEG solution per se. The stability of PEG-car could be as a result of the steric hindrance by PEG which interferes with the interaction between L-carnosine and CN-1. In particular, the PEGylation of L-carnosine leads to a derivative that could not be recognized by the catalytic site of carnosinase, but it is a pity that PEG-car lacks the ability to inhibit enzyme activity.

Against our initial hypothesis, our study showed both PEG-car and L-carnosine had similar pharmacokinetic parameters in serum. This might be principally due to the varied distribution of CN-1, as the expression pattern of *CNDP1* varies in humans and rodents. Human *CNDP1* is expressed extensively in liver and secreted massively in serum, whereas in rodents *CNDP1* is mainly expressed in kidney and is not secreted (Pandya et al. 2011). The absence of serum CN-1 in mice signifies the lack hydrolysis of L-carnosine in serum and thus explaining why pharmacokinetic parameters for PEG-Car and L-carnosine were similar. This is in strong contrast to the kidney where significant differences between both compounds were noticed. Indeed, our data revealed that in kidney the t_1/2_ and Vss of PEG-car were superior than that of L-carnosine. Besides, the comparable pharmacokinetic parameters in serum might also be partly owing to the limitations in our pharmacokinetic work. Although the measurements of PEG-car and L-carnosine in the plasma and kidney were made at different time points, each animal in the study provided a single plasma and kidney sample at each time point. In terms of these pharmacokinetic parameters, our study thereafter combined all the available data in a single analysis by making use of a naïve pooled and sparse sampling approach (Mahmood 2014; KuKanich et al. 2007). Unfortunately, variability cannot be assessed by naïve pooled approaches (KuKanich et al. 2007) which made it difficult to determine the variation of the pharmacokinetic parameters in each animal.

In addition, our data unexpectedly found that the Vss of L-carnosine was greater than that of PEG-car in serum, whilst AUC and the clearance were similar for the two compounds. The reasons for this difference are not clear, however could further be assessed in a dedicated future pharmacokinetic study which would allow a more comprehensive comparison.

Nevertheless, PEG-car was more resistant to CN-1 than L-carnosine in kidney. As a matter of fact, the role of CN-1 and L-carnosine in diabetic kidney diseases has already been verified by plenty of evidences. Diabetic patient with higher serum CN-1 concentration and activity are thought to be more susceptible to DN (Janssen et al. 2005); vice versa, L-carnosine could retard DN development in diabetic mice (Sauerhofer et al. 2010; Qiu et al. 2020). Further studies using human *CNDP1* transgenic ob/ob mice (Qiu et al. 2020) which could express CN-1 in serum and kidney and thereby closely resembles overt human DN would be useful to further explore the efficacy of PEG-car as compared to L-carnosine.

In conclusion, our study for the first time designed and synthesized L-carnosine derivative, PEG-car, and confirmed that PEG-car had strong resistance to CN-1. We also preliminarily evaluated the pharmacokinetics of PEG-car and L-carnosine in mice. h*CNDP1* transgenic mice are warranted anyway to investigate the efficacy of PEG-car as compared to L-carnosine in DN.

## Data Availability

No datasets were generated or analysed during the current study.
